# Barriers and facilitators that hospital clinicians perceive to discuss the personal values, wishes, and needs of patients in palliative care: a mixed-methods systematic review

**DOI:** 10.1177/26323524231212510

**Published:** 2023-12-01

**Authors:** Sita de Vries, Mary-Joanne Verhoef, Sigrid Cornelia Johanna Maria Vervoort, Yvette Milene van der Linden, Saskia Cornelia Constantia Maria Teunissen, Everlien de Graaf

**Affiliations:** Center of Expertise in Palliative Care, Julius Center for Health Sciences and Primary Care, University Medical Center Utrecht, Heidelberglaan 100, P.O. Box 85500, Utrecht 3508 GA, The Netherlands; Center of Expertise in Palliative Care, Leiden University Medical Center, Leiden, The Netherlands; Department of General Practice and Nursing Science, Julius Center for Health Sciences and Primary Care, University Medical Center Utrecht, Utrecht, The Netherlands; Center of Expertise in Palliative Care, Leiden University Medical Center, Leiden, The Netherlands; The Netherlands Comprehensive Cancer Organisation, Utrecht, The Netherlands; Center of Expertise in Palliative Care, Julius Center for Health Sciences and Primary Care, University Medical Center Utrecht, Utrecht, The Netherlands; Center of Expertise in Palliative Care, Julius Center for Health Sciences and Primary Care, University Medical Center Utrecht, Utrecht, The Netherlands

**Keywords:** communication, hospitals, palliative care, patient-centered care, quality of life, systematic review

## Abstract

**Background:**

The exploration and monitoring of the personal values, wishes, and needs (VWN) of patients in the palliative phase by hospital clinicians is essential for guiding appropriate palliative care.

**Objective:**

To explore the barriers and facilitators concerning communication with patients in the palliative phase about their VWN as perceived by hospital clinicians.

**Design:**

A mixed-methods systematic review following the Joanna Briggs Institute guidelines for mixed-method systematic reviews and Preferred Reporting Items for Systematic Reviews and Meta-Analysis (PRISMA) guidelines was conducted (PROSPERO ID: CRD42021216693).

**Data sources and methods:**

Eight databases, including PubMed, Embase, and CINAHL, were searched without time restrictions. The search string was built using the search Palliative cAre Literature rEview iTeraTive mEthod (PALETTE) framework. Eligible studies focused on (1) hospital clinicians and (2) perceived barriers and facilitators regarding the exploration and monitoring of the VWN of adult patients in the palliative phase. Two researchers independently selected articles and evaluated the quality. Findings were synthesized using a convergent integrated approach.

**Results:**

In total, 29 studies were included: 14 quantitative, 13 qualitative, and 2 mixed methods. Five synthesized findings were identified: (1) the clinician’s professional manners, (2) the image formed of the patient and loved ones, (3) the human aspect of being a clinician, (4) the multidisciplinary collaboration, and (5) the contextual preconditions. Most studies seemed focused on communication about treatment decision making.

**Conclusion:**

A patient-centered approach seems lacking when clinicians discuss the patient’s VWN, since most studies focused on treatment decision making rather than on the exploration and monitoring of the multidimensional well-being of patients. This review emphasizes the need for the development and integration of a systematic approach to explore and monitor the patients’ VWN to improve appropriate palliative care in hospitals.

## Introduction

Worldwide, 58 million people are in need of palliative care, and 14% of them currently receive it.^1^ Palliative care focusses on optimizing the quality of life of patients and their loved ones by relieving and preventing suffering from symptoms with multidimensional expressions in the physical, psychological, social, and/or existential dimensions.^2^Appropriate palliative care can be defined as care that fits the personal values, wishes, and needs (VWN) of the patient, while inappropriate palliative care potentially decreases the patient’s quality of life.^3–5^

Palliative care can be provided in a wide range of contexts, such as in hospitals, nursing homes, hospices, or at home. In the last year of their life, patients in the palliative phase are hospitalized on average 2.28 times.^6^ To achieve appropriate palliative care, it is essential to first get to know the person behind the illness by acquiring insight into the personal values: what means the most in life to a person, and what living well means to them.^7–10^ In addition, the patient’s multidimensional wishes and needs should be explored and monitored when the illness progresses, while clinicians respect the patient’s perception of their quality of life.^11^ This exploration and monitoring of VWN supports discussing and organizing appropriate treatment and care.

Since patients in the palliative phase can be admitted to different wards, depending on their current health problem, hospital palliative care is provided by clinicians from a variety of specialisms. Due to a lack of time, competences and uncertainty about responsibilities and roles, VWN often seem neglected in communication between patients and hospital clinicians.^12,13^ Moreover, patients in the palliative phase indicated that communication with hospital clinicians is generally poor because they have difficulties in understanding the language clinicians used and the information provided was not tailored to the patient’s individual preferences.^14^

Communication between hospital clinicians and patients in the palliative phase is suboptimal.^12–14^ Insight into the barriers and facilitators perceived by hospital clinicians is necessary to optimize the exploration, monitoring, and guidance of the patient’s VWN. In this study, barriers and facilitators were defined as the actual and perceived factors, for example, perceptions or beliefs that made it difficult/impossible (barrier) or easy/more likely (facilitator) for clinicians to explore, monitor, and use the patients’ VWN.^15^ Knowing these perceived barriers and facilitators informs the development and implementation of interventions supporting the exploration and monitoring of the patient’s VWN in clinical practice in hospitals. Therefore, this systematic review aimed to explore the barriers and facilitators concerning communication with patients in the palliative phase about their VWN as perceived by hospital clinicians.

## Methods

### Design

A mixed-methods systematic review, following the Joanna Briggs Institute (JBI) methodological guidance for mixed-methods systematic reviews, was performed from September 2020 to March 2023.^16^ Since both qualitative and quantitative methodologies are suitable for exploring the barriers and facilitators, a convergent integrated approach of the JBI was used to synthesize the qualitative and quantitative findings. The Preferred Reporting Items for Systematic Reviews and Meta-Analysis (PRISMA) framework is adhered to for this report in order to enhance the transparency of reporting.^17^ The protocol of this review was prospectively registered in Prospero (ID: CRD42021216693).^18^

### Search strategy and databases

Electronic searches were conducted in PubMed, Web of Science, Embase, Academic Search Premier, COCHRANE Library, CINAHL, Emcare, and PsycINFO, on 23 November 2020 and updated on 27 December 2022. Due to the heterogeneity of palliative care in clinical practice with differences in patient characteristics, stages of illnesses, and the stakeholders involved, the Palliative cAre Literature rEview iTeraTive mEthod (PALETTE) framework^19^ was used to develop a search strategy. In addition, standardized search filters for palliative care were used.^20^ The four phases of the framework were followed. After developing the research question, an initial search was conducted to identify relevant articles which studied the barriers and facilitators concerning communication with patients in the palliative phase about their VWN as perceived by hospital clinicians. Next, four ‘golden bullets’^21–24^ were identified. These golden bullets provided multiple synonyms and keywords. Both were used to optimize the search strategy. This process was conducted in collaboration with a librarian (Jan Schoones). In our final search, all golden bullets were identified, which validated the final search strategy. The final search strategy was constructed using a Domain, Determinant, and Outcome (DDO) outline: Domain: hospital clinicians in palliative care; Determinant: communication about the patient’s VWN; Outcome: barriers and facilitators. No filters or time restrictions were applied in the search. The reference lists of the included articles were screened for additional relevant studies by two reviewers (SdV and M-JV). See Appendix 1 for the complete search string.

### Eligibility criteria

Broad inclusion criteria were used to identify perceived barriers and facilitators related to all forms of communication in the hospital patient trajectory where VWN might be discussed, such as breaking bad news or goals-of-care conversations. Studies were *included* if they met the following criteria:

- Studies from the perspective of hospital clinicians: nurses, nurse practitioners, and medical doctors providing care to adult patients in the palliative phase.
- Studies with barriers and facilitators concerning communication about VWN, such as breaking bad news, prognosis, goals-of-care, advance care planning, and progression to the terminal phase of illness, with patients in the palliative phase as the outcome.
- Studies in English, published in a peer-reviewed journal.

Studies were *excluded* if they met one of the following criteria:

- Studies focused on interns, medical, or nursing students due to the different and mostly educational focus of these studies.
- Studies fully focused on communication in the intensive care unit, primary care, general practice, or long-term care facilities, since this review focused on the care provision to patients in the palliative phase receiving hospital care provided at nursing wars or outpatient clinics.
- Reviews or randomized controlled trials, focused on, for example, new interventions to improve communication.

### Study selection

The results of the searches were imported into Rayyan, a web-based screening program that supports a systematic selection of studies.^25^ Two researchers (SdV and M-JV) independently screened all studies for appropriateness by title and abstract. Next, the full texts were screened based on inclusion and exclusion criteria. There were 21 disagreements about inclusion between the two researchers that were resolved by discussion until consensus was reached. For two studies in which no consensus was reached, a third senior researcher (EdG) was consulted.

### Data extraction

Two data extraction forms were developed by two researchers (SdV and M-JV) – one for the qualitative and one for the quantitative studies – based on examples of the JBI^26^ and the Cochrane collaboration.^27^ Both forms consisted of items concerning the bibliographic information, aim, design, type of clinician, setting, barriers, facilitators, data collection, data analysis, and study outcome of the included studies. The differences between the qualitative and the quantitative form supported a ‘fit to each’ methodology. Both forms were pilot tested on one qualitative and one quantitative study. The outcomes of these pilot tests were discussed by the entire research group (SdV, M-JV, SV, YvdL, ST, and EdG), after which consensus was reached. Two researchers (SdV and M-JV) extracted the data of the included studies and discussed the completed forms, with minor changes or additions made.

### Quality appraisal

The study quality was assessed independently by two reviewers (SdV and M-JV), using the JBI Checklist for Analytical Cross-Sectional Studies^28^ and the JBI Critical Appraisal Checklist for Qualitative Research.^26^ The checklist for cross-sectional studies consisted of eight items concerning the study sample, outcome measurement, confounding, and statistical analysis. The checklist for qualitative studies consisted of ten items concerning the congruity of the study, the role of the researcher, ethical considerations, and the coherence of the conclusion. Both reviewers assessed the individual items of the checklists for each study and discussed their evaluations afterwards. Disagreements were discussed with a third researcher (EdG) until consensus was reached. When a study met the quality requirement, a score of 1 was assigned to the item. When information was unclear, a score of 0.5 was assigned. A score of 0 was assigned to an item if a study did not meet the quality requirement or when information was not provided. Lastly, a final score was calculated for each study. For qualitative studies, this was a maximum final score of 10, for quantitative studies a maximum final score of 8. The final score did not influence inclusion because no studies were excluded based on quality requirements.

### Data transformation and synthesis

Since both qualitative and quantitative studies were included and a convergent integrated approach of the JBI was followed, three steps were performed to analyze the data.^16^ First, the data were extracted: all qualitative themes and all quantitative findings with a percentage <0% were noted. The quantitative findings were ‘qualitized’ by translating and converting findings into a textual description. The ‘qualitized’ findings provided a narrative interpretation of each quantitative finding. All themes and textual descriptions were thereafter combined. Second, all findings were reread so as to become familiar with the data. When the reviewers had sufficient insight into the assembled data, it was categorized into categories with similarity in meaning. Third, categories were thematically aggregated into synthesized findings again based on similarity in meaning. NVivo12 QSR International^29^ was used to assist the analysis. The data transformation and synthesis were performed by three reviewers (SdV, M-JV, and EdG). Preliminary categories and synthesized findings were discussed by the research team (SdV, M-JV, SV, YvdL, ST, and EdG). Small changes were made to the definitions of some categories and synthesized findings to ensure correct interpretation. Theoretical memos were used to record methodological issues and to reflect on the reviewers’ role.^30^

## Results

### Study selection

**Figure 1.**
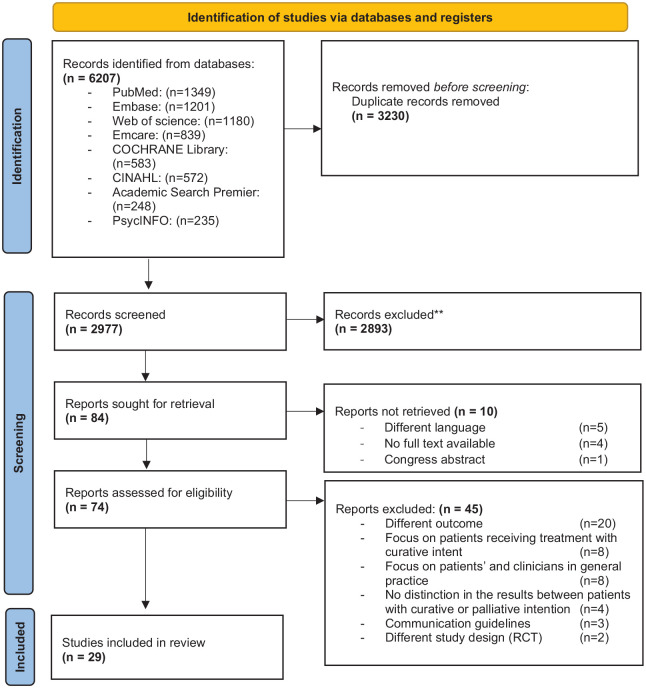
Preferred Reporting Items for Systematic Reviews and Meta-Analysis (PRISMA) diagram.^17^

The search identified 2977 unique studies. After screening on the basis of title and abstract, 2893 articles were excluded. Eighty-four full-text articles were assessed for eligibility, of which 29 were included in this review. Four articles were included after checking the reference lists of the included articles. See Figure 1 for the PRIMSA flowchart.

### Study characteristics

Tables 1 to 3 show the characteristics of the included studies: 14 quantitative, 13 qualitative, and 2 mixed-methods studies, conducted between 2005 and 2022. All quantitative studies had a cross-sectional design and used descriptive statistics. All qualitative studies had a generic qualitative design. In most studies, semi-structured interviews (*n* = 8) or focus groups (*n* = 2) were conducted. Some studies (*n* = 3) used a qualitative analysis for open-ended questions in a survey or after an online education module. One mixed-methods study used both a questionnaire and semi-structured interviews and one mixed-methods Delphi study was included. One mixed-method study only used qualitative data to explore barriers and facilitators and was therefore considered to be a qualitative study.^31^

**Table 1.** Study characteristics of the quantitative studies

Author/year/country	Nature of communication	Setting	Sample	Outcome	Study quality*
You *et al*.^21^/2015/Canada	Mutual disclosure	Internal medicine	512 nurses 484 residents 260 staff physicians	Barriers to communication and decision-making about goals-of-care	7.5/8
Chandar *et al*.^32^/2017/United States	Mutual disclosure	Cardiology, oncology, primary care physicians	35 hematologists/oncologists 29 cardiologists 53 primary care physicians	Barriers to timely ACP	6/8
Fulmer *et al*.^33^/2018/United States	Mutual disclosure	Internists, primary care providers, oncology, cardiology and pulmonology	94 cardiologists 87 pulmonologists 85 oncologists	Views on advanced care planning, goals-of-care, and end-of-life conversations	4.5/8
Oh *et al*.^34^/2019/Canada	Mutual disclosure	Radiation oncology	60 radiation oncologists	Barriers to end-of-life conversations	7/8
Kimura *et al*.^35^/2020/Japan	Mutual disclosure	Oncology	993 nurses 494 oncologists	Barriers to end-of-life discussion	7/8
Spring *et al*.^36^/2021/Canada, United Kingdom	Mutual disclosure	Hematology	58 intensivists 53 hematologic oncologists	Barriers to ACP	6.5/8
Dias *et al*.^37^/2022/Brazil	Mutual disclosure	Oncology	66 oncologists	Barriers to discuss goals-of-care and advance care planning	6/8
Smith *et al*.^38^/2022/Denmark	Mutual disclose	Hospital physicians and general practitioners	622 hospital physicians 160 GPs	Facilitators and barriers for initiating conversations about the end-of-life	7.5/8
Rosenberg *et al*.^39^/2017/United States	Transfer of a message	Hospitalist physicians	253 hospitalists	Barriers around serious illness communication	7.5/8
You *et al*.^40^/2017/Canada	Transfer of a message	Cardiology	469 cardiology nurses 214 staff cardiologists 87 cardiology fellows	The importance of different barriers to communication and decision making about goals-of-care	7.5/8
Ethier *et al*.^24^/2018/United Kingdom	Transfer of a message	Oncology	30 oncologists	Barriers to early goals-of-care discussions	6.5/8
Piggott *et al*.^41^/2019/Canada	Transfer of a message	Oncology	28 nurses 28 oncology residents 10 oncologists	Barriers to goals-of-care discussions	6.5/8
Koh *et al*.^42^/2018/Korea	Transfer of a message	Oncology	229 residents 147 oncologists	Barriers to end-of-life discussions	6.5/8
Cardona *et al*.^43^/2019/Australia	Transfer of message	Emergency, intensive care, or palliative care	360 nurses and physicians	Barriers to and enablers for the end-of-life discussions	7.5/8

ACP, advance care planning; GPs, general practitioners.

* JBI Checklist for Analytical Cross-Sectional Studies.

**Table 2.** Study characteristics of the qualitative studies.

Author/year/country	Nature of communication	Setting	Sample	Outcome	Data collection	Study quality*
Nouvet *et al*.^44^/2016/Canada	Mutual disclosure	Wards for patients with non-surgical serious illness	18 physicians 12 nurses	Barriers related to communication and decision making about goals-of-care	30 semi-structured interviews	9/10
Booker *et al*.^45^/2018/Canada	Mutual disclosure	Hematology	4 physicians 3 nurse practitioners 1 social worker	Barriers to and facilitators of ACP	8 semi-structured interviews	8/10
Bergenholtz *et al*.^22^/2019/Denmark	Mutual disclosure	Pulmonary medicine and surgery	6 physicians 5 nurses 3 social and healthcare assistants	Existing practices regarding EOL conversations	66 h participant observations 4 homogeneous focus groups	8/10
Argintaru *et al*.^46^/2019/Canada	Mutual disclosure	Emergency department	108 EM physicians 23 EM residents	Barriers and facilitators to conducting goals-of-care discussions	Online survey with a qualitative analysis of one open-ended question	7/10
Ladin *et al*.^47^/2021/United States	Mutual disclosure	Nephrology	22 nephrologists 4 physician assistants	Barriers to ACP	26 semi-structured interviews	9/10
LoCastro *et al*.^48^/2022/United States	Mutual disclosure	Hematology	16 oncology clinicians 9 palliative care clinicians	Challenges to ACP	25 semi-structured interviews	7/10
Devery *et al*.^49^/2022/Australia	Mutual disclosure	Hospital settings	587 nurses 226 physicians 128 allied health professionals	Challenges to negotiating goals-of-care at the end-of-life	Qualitative analysis of one open-ended question in an online education module	8/10
Anselm *et al*.^23^/2005/United States	Transfer of a message	Internal medicine	33 nurses 24 residents 10 physicians	Barriers to end-of-life discussions	11 homogeneous focus groups	7/10
Granek *et al*.^50^/2013/Canada	Transfer of a message	Oncology	20 oncologists	Barriers to discussing end-of life issues	20 semi-structured interviews	8/10
Banerjee *et al*.^51^/2015/United States	Transfer of a message	Oncology	121 nurses	Communication challenges related to end-of-life care issues	Online survey with a qualitative analysis of two open-ended questions	8/10
Schulman-Green *et al*.^52^/2018/United States	Transfer of a message	Oncology	21 oncologists	Facilitators of and barriers to goals-of-care conversations	21 semi-structured interviews	9/10
Levinson *et al*.^53^/2019/Australia	Transfer of a message	Emergency department	14 emergency doctors 4 internal medicine specialists	Opinions and how doctors undertake goals-of-care conversations.	18 semi-structured interviews	9/10
Diendorfer *et al*.^31^/2022/Austria	Transfer of a message	Oncology	44 oncologists	Limiting and supporting factors of end-of-life communication	44 semi-structured interviews	9/10

ACP, advance care planning; EOL, end-of-life; GPs, general practitioners.

* JBI Critical Appraisal Checklist for Qualitative Research.

**Table 3.** Study characteristics of the mixed-methods studies.

Author/year/country	Nature of communication	Setting	Sample	Outcome	Data collection	Data analysis	Study quality*,**
Vanderhaeghen *et al*.^54^ **/**2019**/**Belgium	Mutual disclosure	Hospital professionals considered to have ACP conversations	Round I: 11 nurses, 5 physicians, 5 paramedics (psychologists/social workers) Round II: 9 nurses, 5 physicians, 5 paramedics (psychologists/social workers)	Obstacles and helping factors for having ACP conversations	The Delphi survey technique: series of questionnaires Round I: Open-ended questions. Round II: The second questionnaire was built on the responses to the first	Content analysis and descriptive statistics	8/10* 6.5/8**
Periyakoil *et al*.^55^ **/**2015**/**United States	Transfer of a message	Multi-specialty doctors who care for seriously ill patients	1040 multi-specialty doctors: 289 internal medicine, 188 surgery, 140 pediatrics, 95 anesthesiology, 74 radiation and nuclear medicine, 52 psychiatry, 50 pathology, 32 neurology, 29 emergency medicine, 25 obstetrics and gynecology, 22 physical medicine and rehabilitation	Doctor-reported barriers to end-of-life conversations	Questionnaire with closed and open-ended questions	Qualitative data analyses of development cohort to identify key codes	7/10* 4/8**

ACP, advance care planning.

* JBI Critical Appraisal Checklist for Qualitative Research.

** JBI Checklist for Analytical Cross-Sectional Studies.

Most studies were conducted in the United States (*n* = 9) and Canada (*n* = 9). The other studies were conducted in Australia (*n* = 2), Denmark (*n* = 2), the United Kingdom (*n* = 2), Belgium, Japan, Austria, Brazil, and Korea. In 13 studies, clinicians provided care to patients with advanced cancer. In nine studies, clinicians of various specialisms were included. In total, 4249 physicians, 2781 nurses, 770 residents, 7 nurse practitioners, and 142 paramedics or social workers participated.

The mean quality score of the included quantitative and qualitative were quantitative: 6.7/8 (range: 4/8–7.5/8) and qualitative: 8.2/10 (range: 7/10–9/10), respectively. For the mixed-methods studies were both the checklist for analytic cross-sectional studies and the checklist for qualitative research used. The mean score for mixed-methods studies was: 12.74/18 (range:11/18–14.5/18). In all quantitative studies, a self-developed questionnaire was used with limited validity testing. Almost all studies explored face and/or content validity. In most qualitative studies, the reflexivity analysis was provided to a limited degree or was lacking. The role of the researcher was typically not elaborated. Furthermore, the philosophical or theoretical foundations of the studies and/or the methodological approaches were provided to a limited extent or, in some instances, not provided at all (Tables 4 to 6).

**Table 4.** Quality appraisal of the quantitative studies.

	You *et al.*^21^	Chandar *et al*.^32^	Fulmer *et al*.^33^	Oh *et al.*^34^	Kimura *et al.*^35^	Spring *et al*.^36^	Dias *et al*.^37^	Smith *et al.*^38^	Rosenberg *et al*.^39^	You *et al.*^40^	Ethier *et al*.^24^	Piggot *et al.*^41^	Koh *et al.*^42^	Cardona *et al*.^43^
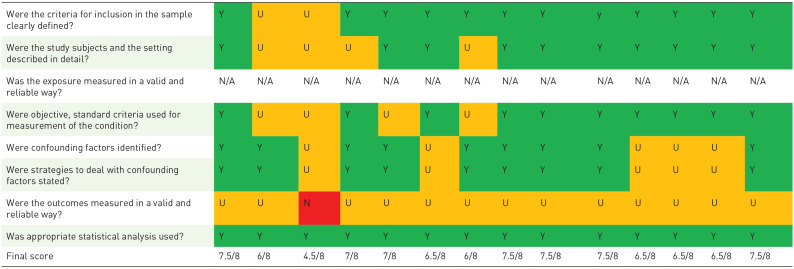														

Y, yes; N, no; U, unclear; N/a, not applicable correspond to these color.

**Table 5.** Quality appraisal of the qualitative studies.

	Nouvet *et al*.^44^	Booker *et al.*^45^	Bergenholtz *et al*.^22^	Argintaru *et al*.^46^	Ladin *et al*.^47^	LoCastro *et al*.^48^	Devery *et al.*^49^	Anselm *et al*.^23^	Granek *et al*.^50^	Banerjee *et al.*^51^	Schulman-Green *et al.*^52^	Levinson *et al*.^53^	Diendorfer *et al.*^31^
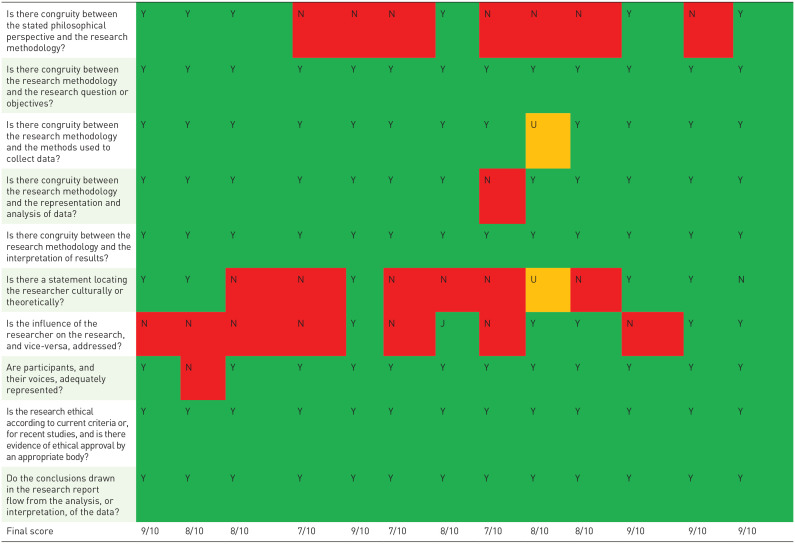													

N, no; N/a, not applicable; U, unclear; Y, yes.

Y, yes; N, no; U, unclear; N/a, not applicable correspond to these color.

**Table 6.** Quality appraisal of the mixed-methods studies.

	Vanderhaeghen *et al*.^54^ (1)^a^	Periyakoil *et al*.^55^ (2)^b^		Vanderhaeghen *et al*.^54^ (3)^c^	Periyakoil *et al*.^55^ (4)^d^
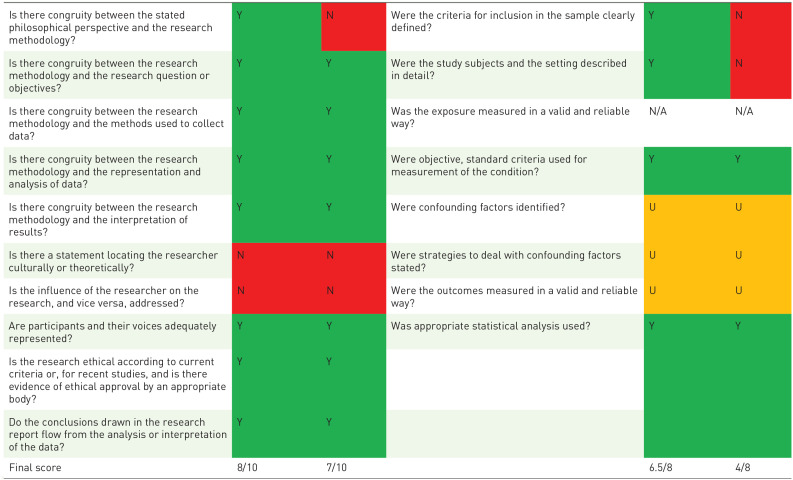					

N, no; N/A, not applicable; U, unclear; Y, yes.

a Vanderhaeghen *et al*. (1) consider the qualitative arm of the study

b Periyakoil *et al*. (2) consider the qualitative arm of the study

c Vanderhaeghen *et al*. (3) consider the quantitative arm of the study

d Periyakoil *et al*. (4) consider the quantitative arm of the study.

Y, yes; N, no; U, unclear; N/a, not applicable correspond to these color.

### Definition of communication about VWN

Communication about the VWN provided in the included studies was in the context of breaking bad news, goals-of-care, advance care planning, and disease progression to the dying phase. Based on the included articles, two types of communication were distinguished in the study characteristics: communication including mutual disclosure between the patient and clinicians about the patient’s VWN (*n* = 16) and communication ensuring the transmission of a message from the clinician to the patient (*n* = 13), for example, treatment restrictions, illness progression, or a limited life expectancy.

### Data synthesis

The barriers and facilitators concerning communication with patients in the palliative phase about their VWN as perceived by hospital clinicians were analyzed, after which five synthesized findings were identified: (1) the clinician’s professional manners, (2) the image formed of the patient and loved ones, (3) the human aspect of being a clinician, (4) the multidisciplinary collaboration, and (5) the contextual preconditions. These findings were identified from 23 categories based on 305 unique findings: 218 quantitative and 87 qualitative. Each category could contain multiple unique findings from one study. It is important to note that ethical, legal, and financial findings were excluded in this synthesis since these differ culturally per country and therefore cannot be merged into one synthesized finding. Findings that made it difficult or impossible to discuss the patient’s VWN were seen as a barrier, and those that made it easy or more likely were seen as a facilitator. All findings represent both barriers and facilitators, as these are poles of the same continuum.

#### Synthesized finding 1: The clinicians’ professional manners

**Figure 2.**
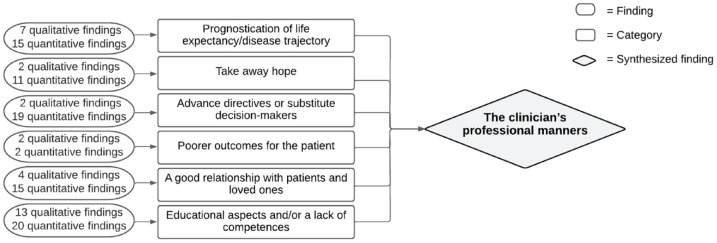
Synthesized finding 1: The clinician’s professional manners.

Several aspects related to the clinician’s professional manners were perceived as a barrier. Figure 2 shows which categories of barriers and facilitators constitute this synthesized finding. A conversation about VWN follows after the identification of the palliative care needs. *Prognostication of life expectancy/disease trajectory*^21,23,24,33–35,37,38,40–44,46,47,49,50^ is essential to an appropriate identification. The predictability of the disease trajectory influenced the self-confidence of clinicians to initiate a conversation. Finding the right time to start a conversation about VWN was viewed as difficult by clinicians. In addition, clinicians were worried that conversations about VWN could *take away hope*^21,24,33–37,40–42,45,53^ in patients and loved ones and when they wanted to maintain a positive perspective. Clinicians usually had their own routines for maintaining hope. The presence of *advance directives or substitute decision makers*^21,23,24,37,40,41,43,46^ were seen as facilitators. If a patient’s record included an advance directive with preferences and decisions about care and treatment provision and limitations on care and treatment, clinicians find it easier to monitor this previously discussed information. In four studies, clinicians were concerned that speaking about VWN could *lead to poorer outcomes for the patient*.^23,31,34,54^ Additionally, clinicians considered *a good relationship with patients and loved one*^21,22,24,31,34,36–42,46,55^ to be both a facilitator and a barrier to a conversation about VWN. Clinicians expected that discussing VWN with patients would harm a good relationship, which they find undesirable. Clinicians who did not have a long relationship with the patient or did not know the patient well found it more difficult to initiate a conversation about VWN than those who knew their patient well or for a longer time. *Educational aspects and/or a lack of competence*^21–24,31,32,34–43,46,47,49–54^ were addressed as barriers or facilitators in several studies. Clinicians stated that training and communicational skills were essential when communicating about VWN with a patient. A lack of competence due to inadequate or no training was perceived as a barrier that gave clinicians an uncomfortable feeling when speaking with patients about their VWN. Mentorship, in the form of watching supervisors, seemed to be helpful for gaining experience.

#### Synthesized finding 2: The image formed of the patient and loved ones

**Figure 3.**
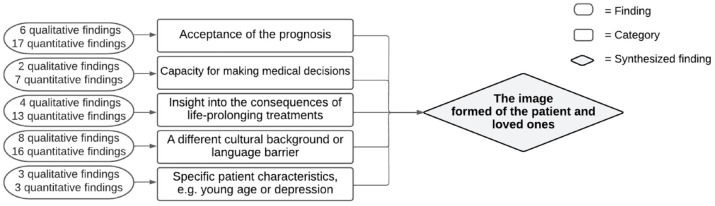
Synthesized finding 2: The image formed of the patient and loved ones.

Clinicians seemed to form an image of patients and their loved ones that was not explicitly validated with them. Figure 3 shows the construction of this synthesized finding. The imaging of patients and loved ones mostly influences the initiation of a conversation about VWN negatively. First, clinicians took into account their estimation of the patient and loved ones’ *acceptance of the prognosis.*^21,24,35–38,40,41,49–52^ Difficulties in accepting a prognosis and/or negative reactions from loved ones could hinder a clinician from initiating a conversation about VWN. Next, clinicians estimated whether patients and loved ones *were able to make decisions about treatment*^21,24,35,37,40,41,43,46^ and whether patients and their loved ones could *oversee the consequences of life-prolonging treatments*^21–24,35–37,40,41,45,52^ in terms of side effects or complications. When clinicians estimated that a patient had difficulties in decision making or overseeing side effects and complications, this complicated the initiation of a conversation about VWN. In addition, *a different cultural background or language barrier*^21,23,24,31,33,34,37,39–41,43,46,50,52,55^ could complicate conversations. Clinicians perceived cultural or language barriers as problematic as they felt they could not understand the patient and loved ones correctly and vice versa. Finally, *specific patient characteristics*^24,31,37,50,51,54,55^ could influence discussing VWN with, for example, young patients or the patient’s high health literacy. See Appendix 2 for an overview of the specific patient characteristics.

#### Synthesized finding 3: The human aspect of being a clinician

**Figure 4.**
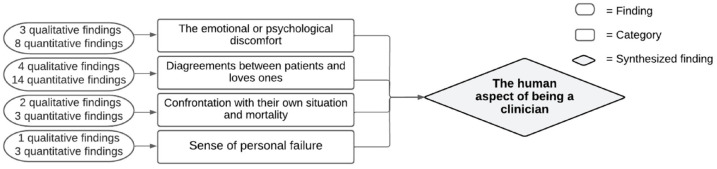
Synthesized finding 3: The human aspect of being a clinician.

The human aspect of being a clinician relates to the confrontation of clinicians with the reactions and emotions of patients and loved ones that could affect a clinician personally. Figure 4 shows how this synthesized finding is constructed. Clinicians were confronted with *the emotional or psychological discomfort of patients and loved ones*,^23,24,32–34,36,39,48,49^ making them feel uncomfortable. The emotions and psychological discomfort of patients and loved ones requires a compassionate response in which clinicians need to be able to continue to act as is expected in their role as a clinician. Also, *disagreements between the patient and loved ones or between loved ones*^21,23,24,33,35–37,39–43,49,50^ made it more difficult for clinicians to discuss the patient’s VWN. This also applies to the wish or request of loved ones not to involve the patient in a conversation or a loved one’s wish to continue the patient’s treatment. Moreover, discussing VWN with patients in the palliative phase *could confront clinicians with their own situation and mortality*.^37,42,50,51^ In four studies, clinicians viewed it as a *sense of personal failure*^34,42–44^ when a patient could no longer be cured of illness. The categories created discomfort for clinicians, which was handled differently. Clinicians could not always find the right words to initiate a conversation about VWN. Other clinicians preferred to avoid the conversation and strong emotions, or preferred that a colleague spoke with the patient about their VWN. Others used euphemisms or positive language when discussing treatment side effects with patients.

#### Synthesized finding 4: The multidisciplinary collaboration

**Figure 5.**
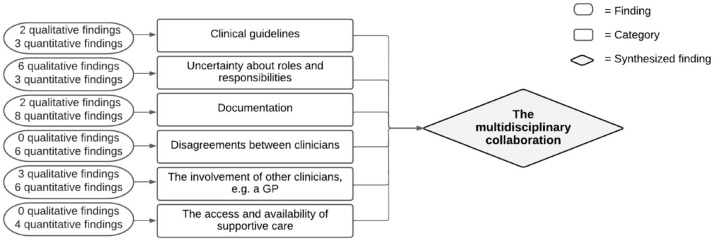
Synthesized finding 4: The multidisciplinary collaboration.

The multidisciplinary collaboration affected clinicians in their communication with patients about their VWN in several areas. The construction of this synthesized finding is depicted in Figure 5. In some of the studies included, clinicians indicated that they were not aware of *clinical guidelines*^35,43,45,50,54^ concerning palliative care and communication about the patient’s VWN or that these guidelines were lacking. Each clinician applied their own routine and timing in discussing VWN. This led to *uncertainty about roles and responsibilities*.^23,34,36,39,44,47,50,51^ It was not always clear to clinicians who was the right person to discuss the patient’s VWN. This applied to the different types of clinicians in the hospital. For example, clinicians were unsure whether the physician or nurse was the right person to initiate a conversation about VWN, or perhaps a clinician who was involved throughout the entire patient trajectory, such a general practitioner. In addition, when VWN were discussed, this was *often documented to a limited degree or not documented at all*^21,23,24,35,37,39–41,47^ in the patient’s medical record. This limited the opportunities for clinicians to follow-up on and monitor previously discussed topics, specifically when various clinicians were involved. Moreover, *disagreements between clinicians*^21,24,37,39–41^ about the patient’s goals-of-care were seen as a barrier to discussing VWN with the patient because then clinicians did not know what was correct to discuss. *The involvement of other clinicians*^35,38,39,43,45,50,52,54^ facilitated clinicians in discussing VWN. Other clinicians consulted a colleague in the hospital with expertise in palliative care or a general practitioner who knew the patient’s situation well. *The lack of access and availability of supportive care*^35,36,43^ for the patient, for example, social work, psychologic support, or chaplaincy, was perceived as a barrier in three studies and hindered a conversation about the patient’s VWN.

#### Synthesized finding 5: The contextual preconditions

**Figure 6.**
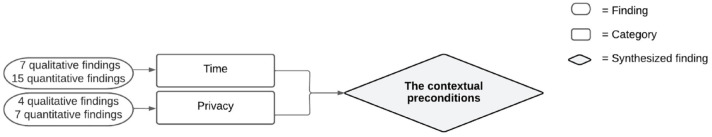
Synthesized finding 5: The contextual preconditions.

The contextual preconditions were seen as conditions to discuss VWN. Figure 6 shows how this synthesized finding is constructed. Two categories constituted this finding. *A lack of time*^21–24,32–43,46–48,51,52,54^ was seen as an important barrier for discussing the patient’s VWN. Clinicians also indicated that a location with sufficient *privacy*^21,22,24,31,35,37,38,40,41,46,51^ was an essential precondition. Some studies indicated that clinicians did not start a conversation about VWN when they knew there was not enough time or when there was no location available with enough privacy.

## Discussion

This systematic review aimed to explore the barriers and facilitators concerning the communication with patients in the palliative phase about their VWN as perceived by hospital clinicians. Five synthesized findings were identified: (1) the clinician’s professional manners, (2) the image formed of the patient and loved ones, (3) the human aspect of being a clinician, (4) the multidisciplinary collaboration, and (5) the contextual preconditions.

### Clinician–patient communication and hope

Many clinicians found it difficult to discuss the patient’s VWN at the right time and were also afraid of taking away hope when speaking with the patient about their situation and VWN. The importance of hope in palliative care is acknowledged clearly.^56^ However, hope should be seen as a process in the person’s inner being and is not determined by outside factors but evolves over time, depending on the patient and the situation.^57^ Hope is broader than an expectation that should be in line with the truth. For patient’s in the palliative phase, hope can also be a form of coping or a meaning, and both perspectives should be helpful or valuable for patient’s.^58^ Talking about the patient’s VWN, therefore, seems not to affect hope but, rather, offers clinicians opportunities to guide appropriate palliative care because talking about the patient’s VWN provides insight into what is important for the patient.^59^

Moreover, clinicians seemed to be aware of the need for optimal communication. However, the perceived barriers and facilitators in clinical practice showed a different perspective, indicating that less attention was paid to the exchange and tailoring of information between clinicians and patients. This corresponds to studies focused on patients’ experiences of communication. Patients indicated that communication with clinicians during their hospital admission was often limited due to difficulties in understanding the (medical) language used, a lack of skills, and the amount and type of information that was provided.^14,60,61^ Findings showed that clinicians formed an image of the patient and loved ones, which was not discussed regularly. Transferring a message to the patient and loved ones seemed to be an important result of communication in almost half of the studies included. This is not in line with clinical guidelines and recommended communication strategies for healthcare professionals in end-of-life communication.^62,63^ Strategies described were a process of preparation, exploration, and assessment of the patient’s thoughts and needs, reframing goals-of-care, and the closure of conversations with patients approaching the end of their life.^63^ During a dialog, clinicians should respond empathetically, tailor information to the understanding of the patient, check whether the information was understood correctly, and involve the patient’s loved ones.^63^ These recommended communication strategies are an example of a patient-centered approach, whereas the barriers actually perceived in clinical practice seem to be an example of an applied biomedical approach.^9^ Where a patient-centered approach focuses on the patient as a person and is responsive to the patient’s VWN, the biomedical approach has an illness-orientated focus and is orientated on physical symptoms in particular.^10^ A patient-centered approach is associated with higher patient satisfaction and is reflected in the recommended strategies and clinical guidelines.^62,64^

The differences between the recommended communication strategies and perceived barriers and facilitators could be explained by the various clinicians and the different care settings involved in the patient trajectory. Due to the leading biomedical-oriented approach and focus on medical treatment in the hospital setting, VWN could be discussed more explicitly by clinicians in other care settings, with the general practitioner given a central role.^65^ Coordination between the different types of clinicians, for example, a nurse or physician, with regard to the patient and the settings in the patient trajectory are often limited.^66–68^ Therefore, each clinician in the patient trajectory has a responsibility to explore and monitor patients’ VWN. Clinicians should empower patients by involving them in their care and tailoring decision making to support the VWN over time rather than assuming a passive patient role in a paternalistic relationship by transferring information to them.^69^ Since documentation of these discussions is vital in ensuring continuity of care and dialog, ideally this should be included in informative letters to other clinicians when patients are transferred.^68,70^

### Implications for clinical practice and future research

Although barriers were identified, our results did not clarify whether these barriers withhold clinicians from discussing the patient’s VWN in clinical practice. However, there was no systematic attention paid to the exploration and monitoring of the multidimensional well-being of the patient. In addition to the biomedical approach, a better understanding of the patient’s VWN in the psychological, social, and spiritual dimensions is desirable in order to guide appropriate palliative care.^2,4^ Communication interventions for clinicians who are not specialized in palliative care or have limited experience are primarily focused on breaking bad news. Interventions that would support communication in order to explore and monitor the patient’s VWN, in addition to the bad news message, are scarcely reported.^71^ As a result, the development and integration of a systematic approach as part of regular care is needed. This approach should elaborate on the clinicians’ roles, responsibilities, and agreements on documentation concerning the exploration and monitoring of the patient’s multidimensional VWN.^72^ To ensure that on-going attention is paid to the patients’ VWN, the integration of working methods like palliative reasoning support clinicians to systematically assess patients’ VWN.^73^ The palliative reasoning methodology consists of four phases: (1) map in addition to the physical status the patient’s emotional, socio-spiritual situation^74^; (2) summarize problems and develop a proactive care plan regarding the prognosis of remaining lifetime; (3) make agreements on care plan evaluation; and (4) adjust the care plan and communication when required and continue to evaluate.^75^ However, the integration of such a working method undeniably also requires a change in the culture of collaboration and the clinician’s behavior and attitude.^76^ Since clinicians have the responsibility to give patients the opportunity to address and discuss what matters to them, it is essential to employ open-ended questions in these discussions, extending the conversation beyond treatment wishes and limitations.^77^ Training and education should therefore be an important part of the approach and should be applied during the implementation.^78,79^ A systematic approach to explore and monitor the patient’s VWN will initially probably increase the clinician’s workload temporarily, but ultimately this results in an essential contribution to providing appropriate palliative care. Future research is required to develop the systematic approach to discuss the patient’s VWN alongside guidance by patient participation in the development of research questions and study protocol. Starting with patient-driven qualitative research to gain insight into patients’ experiences and preferences regarding the exploration and monitoring of their VWN.

## Strengths and limitations

A strength of this review was the use of a convergent integrated approach. As a result, all existing research, both qualitative and quantitative methodologies, was merged to provide an overview of all barriers and facilitators without losing any relevant findings.^16^ In addition, the qualitative findings provided more in-depth information, which allowed a better interpretation of the quantitative findings. Another strength of this review was the close cooperation of members of the entire interprofessional research team (SdV, M-JV, SV, YvdL, ST, and EdG). All essential steps in conducting this review were performed by two researchers independently to ensure precision (SdV and M-JV), with a senior researcher (EdG) available for additional consultation. A few limitations must be taken into consideration. The first limitation was the absence of involvement of patients in the development and execution this review. Patient participation should be stimulated to guide future research in this area in order to ensure that future research meets the priorities of patients and is relevant for them.^80^ A second limitation was that the majority of the studies included were conducted in the United States and Canada (*n* = 18). As a result, our results could be affected by the U.S. and Canadian palliative care guidelines and standards. However, we did not find any major differences with the results of the studies performed in other countries (*n* = 11). Finally, it was our intention to include only studies focused on nurses, nurse specialists, and physicians working in hospitals. However, some studies included participation by paramedics or, in addition to hospital clinicians, clinicians working in intensive care or primary care. This pertained to a few studies and a small number of clinicians, in which the results aligned with the included studies that focused on physicians, nurse practitioners, and nurses working in hospitals. These studies were included as well to ensure that no relevant information was lost.

## Conclusion and practical implications

The barriers and facilitators that hospital clinicians perceived in the communication with patients in the palliative phase about their VWN related to the clinician’s professional manners, the image formed of the patient and loved ones, the human aspect of being a clinician, the multidisciplinary collaboration, and the contextual preconditions. The exploration and monitoring of VWN are primarily focused on treatment wishes and limitations while mutual agreements on the clinicians’ roles and responsibilities are lacking. Our findings emphasize the need for the development and integration of a systematic approach to improve on-going attention for the patients’ VWN in clinical practice and translation of these VWN into individualized care during the patient trajectory. Clinicians should take their responsibility to create opportunities for patients to voice their VWN. Moreover, the skillful use of open-ended questions in these conversations, which should extend beyond treatment wishes and limitations, is deemed crucial for acquiring a comprehensive insight into the patient’s VWN. This contributes to optimizing appropriate palliative care, thereby enhancing the patient’s quality of life.

## Appendix

**Appendix 1.** Search string.

Domain	(‘Health Personnel’[majr] OR ‘Healthcare provider*’[tiab] OR ‘health care provider*’[tiab] OR ‘healthcare professional*’[tiab] OR ‘health care professional*’[tiab] OR ‘clinician*’[tiab] OR ‘doctor*’[tiab] OR ‘medical doctor*’[tiab] OR ‘physician*’[tiab] OR ‘resident*’[tiab] OR ‘nurse specialist*’[tiab] OR ‘nurse*’[tiab] OR ‘specialized nurse*’[tiab] OR ‘specialised nurse*’[tiab] OR ‘physician assistant*’[tiab] OR ‘palliative care consultant*’[tiab] OR ‘nurse practitioner*’[tiab])
	AND
	(‘Hospitals’[majr] OR ‘hospital’[tiab] OR ‘hospitals’[tiab] OR ‘hospital-based’[tiab] OR ‘hospitalized’[tiab] OR ‘hospitalised’[tiab] OR ‘Inpatients’[majr] OR ‘in-patient’[tiab] OR ‘in-patients’[tiab] OR ‘inpatient’[tiab] OR ‘inpatients’[tiab] OR ‘ward’[tiab] OR ‘wards’[tiab] OR ‘clinic’[tiab] OR ‘clinics’[tiab] OR ‘inhospital’[tw] OR ‘inhospital’[tw])
	AND
	(‘Palliative Care’[majr] OR ‘Palliative care’[tiab] OR ‘Terminal Care’[majr] OR ‘terminal care’[tiab] OR ‘end-of-life care’[tiab] OR ‘terminal phase’[tiab] OR ‘palliative phase’[tiab] OR ‘dying’[tiab] OR ‘dying phase’[tiab] OR ‘Death’[majr:NoExp] OR ‘death’[tiab] OR ‘imminent death’[tiab] OR ‘palliative’[tiab] OR ‘palliat*’[tiab] OR ‘terminal’[tiab] OR ‘end-of-life’[tiab] OR ‘end of life’[tiab] OR ‘advanced disease’[tiab] OR ‘advanced illness’[tiab] OR ‘seriously ill’[tiab] OR ‘serious illness’[tiab] OR ‘terminally ill’[tiab] OR ‘hospice care’[mesh] OR ‘hospices’[mesh] OR ‘hospice and palliative care nursing’[mesh] OR ‘hospice*’[tiab] OR ‘bereavement care’[tiab] OR ‘hospice program*’[tiab] OR ‘life limiting illness’[tiab] OR ‘life limiting’[tiab] OR ‘lifelimiting’[tiab]) NOT ((‘Child’[mesh] OR ‘Infant’[mesh] OR ‘Adolescent’[mesh]) NOT ‘Adult’[mesh])
AND	
Determinant	(‘Communication’[majr] OR ‘Communication’[tiab] OR ‘communicate’[tiab] OR ‘communicating’[tiab] OR ‘contact’[tiab] OR ‘conversation’[tiab] OR ‘advice’[tiab] OR ‘advise’[tiab] OR ‘advisement’[tiab] OR ‘converse’[tiab] OR ‘correspondence’[tiab] OR ‘corresponding’[tiab] OR ‘express’[tiab] OR ‘expression’[tiab] OR ‘expressing’[tiab] OR ‘intercommunication’[tiab] OR ‘mention’[tiab] OR ‘mentioning’[tiab] OR ‘talk’[tiab] OR ‘talking’[tiab] OR ‘discuss’[tiab] OR ‘discussing’[tiab] OR ‘discussion’[tiab] OR ‘tell’[tiab] OR ‘telling’[tiab] OR ‘notifying’[tiab] OR ‘disclosing’[tiab] OR ‘announcing’[tiab] OR ‘announcement’[tiab] OR ‘Decision Making, Shared’[majr] OR ‘shared decision-making’[tiab] OR ‘Advance Care Planning’[majr] OR ‘advance care planning’[tiab] OR ‘compassion’[tiab] OR ‘compassionate’[tiab] OR ‘Physician-Patient Relations’[majr] OR ‘Professional-Patient Relations’[majr] OR ‘Nurse-Patient Relations’[majr] OR ‘patient-physician relationship’[tiab] OR ‘physician-patient relationship’[tiab] OR ‘doctor-patient communication’[tiab] OR ‘clinician-patient relationship’[tiab] OR ‘clinician-patient communication’[tiab] OR ‘Professional Role’[majr] OR ‘role’[tiab] OR ‘patient-physician communication’[tiab] OR ‘patient-clinician relationship’[tiab] OR ‘patient-clinician communication’[tiab] OR ‘tailored discussion*’[tiab] OR ‘tailored’[tiab] OR ‘tailoring’[tiab] OR ‘tailor’[tiab] OR ‘goals-of-care discussion*’[tiab] OR ‘address*’[tiab] OR ‘Disclosure’[majr] OR ‘dialog*’[tiab] OR ‘sharing information‘ [tw] OR ‘share information’[tw] OR ‘shared information’[tw] OR ‘physician-patient communication’[tw] OR ‘nurse-patient interaction’[tw])
	AND
	(‘Personalized care’[tw] OR ‘personalised care’[tw] OR ‘Patient-Centered Care’[mesh] OR ‘person-centred care’[tw] OR ‘person-centered care’[tw] OR ‘person-centered medicine’[tw] OR ‘person-centred medicine’[tw] OR ‘person-focused care’[tw] OR ‘people-centred care’[tw] OR ‘people-centered care’[tw] OR ‘patient-centered care’[tw] OR ‘patient-centred care’[tw] OR ‘person-centred’[tw] OR ‘person-centered’[tw] OR ‘patient-centered’[tw] OR ‘patient-centred’[tw] OR ‘narrative-based medicine’[tw] OR ‘narrative medicine’[tw] OR ‘Patient Navigation’[tw] OR ‘values’[tw] OR ‘wishes’[tw] OR ‘Patient Preference’[mesh] OR ‘preferences’[tw] OR ‘patient preferences’[tw] OR ‘quality of life’[tw] OR ‘Quality of Life’[mesh] OR ‘personal values’[tw] OR ‘personal value’[tw] OR ‘goals-of-care’[tw] OR ‘preferences for end-of-life care’[tw] OR ‘Cultural Characteristics’[mesh])
AND	
Outcome	(‘Professional Competence’[majr] OR ‘Clinical Competence’[majr] OR ‘Competence’[tiab] OR ‘competencies’[tiab] OR ‘proficiency’[tiab] OR ‘skill*’[tiab] OR ‘ableness’[tiab] OR ‘ability’[tiab] OR ‘able’[tiab] OR ‘capability’[tiab] OR ‘capabilities’[tiab] OR ‘facilitator*’[tiab] OR ‘barrier*’[tiab] OR ‘need*’[ti] OR ‘practice*’[tiab])

**Appendix 2.** Specific patient characteristics – synthesized finding 2.

Barrier	Facilitator
Young patients^50–52^	Older patients^38^
Patient/family’s limited health literacy^55^	Patient’s high health literacy^52^
Patient with young children^51,52^	Patient’s poor functional status^52^
Current patient presentation: for example, patient is ill from an acute, potentially reversible etiology such as infection *versus* decline from disease progression^24,37^	
Mental capacity of a patient: for example, depression, information processing speed or aggression^31^	

## Declarations

**Ethics approval and consent to participate:** Not applicable.

**Consent for publication:** All authors reviewed and approved the final manuscript.

**Availability of data and materials:** The data that support the findings of this review are available from the corresponding author upon request.
